# Gestational Exposure to Bisphenol A Affects the Function and Proteome Profile of F1 Spermatozoa in Adult Mice

**DOI:** 10.1289/EHP378

**Published:** 2016-07-06

**Authors:** Md Saidur Rahman, Woo-Sung Kwon, Polash Chandra Karmakar, Sung-Jae Yoon, Buom-Yong Ryu, Myung-Geol Pang

**Affiliations:** Department of Animal Science and Technology, Chung-Ang University, Anseong, Republic of Korea

## Abstract

**Background:**

Maternal exposure to the endocrine disruptor bisphenol A (BPA) has been linked to offspring reproductive abnormalities. However, exactly how BPA affects offspring fertility remains poorly understood.

**Objectives:**

The aim of the present study was to evaluate the effects of gestational BPA exposure on sperm function, fertility, and proteome profile of F1 spermatozoa in adult mice.

**Methods:**

Pregnant CD-1 mice (F0) were gavaged with BPA at three different doses (50 μg/kg bw/day, 5 mg/kg bw/day, and 50 mg/kg bw/day) on embryonic days 7 to 14. We investigated the function, fertility, and related processes of F1 spermatozoa at postnatal day 120. We also evaluated protein profiles of F1 spermatozoa to monitor their functional affiliation to disease.

**Results:**

BPA inhibited sperm count, motility parameters, and intracellular ATP levels in a dose-dependent manner. These effects appeared to be caused by reduced numbers of stage VIII seminiferous epithelial cells in testis and decreased protein kinase A (PKA) activity and tyrosine phosphorylation in spermatozoa. We also found that BPA compromised average litter size. Proteins differentially expressed in spermatozoa from BPA treatment groups are known to play a critical role in ATP generation, oxidative stress response, fertility, and in the pathogenesis of several diseases.

**Conclusions:**

Our study provides mechanistic support for the hypothesis that gestational exposure to BPA alters sperm function and fertility via down-regulation of tyrosine phosphorylation through a PKA-dependent mechanism. In addition, we anticipate that the BPA-induced changes in the sperm proteome might be partly responsible for the observed effects in spermatozoa.

**Citation:**

Rahman MS, Kwon WS, Karmakar PC, Yoon SJ, Ryu BY, Pang MG. 2017. Gestational exposure to bisphenol-A affects the function and proteome profile of F1 spermatozoa in adult mice. Environ Health Perspect 125:238–245; http://dx.doi.org/10.1289/EHP378

## Introduction

Endocrine disruptors (EDs) are synthetic chemicals that interfere with hormone biosynthesis and metabolism, thereby affecting the body’s endocrinological homeostasis. Exposure to EDs has been linked with developmental, systemic, reproductive, neurological, and immune disorders in both humans and animals (Heindel et al. 2015; Trasande et al. 2015). Studies in human subjects consistently demonstrate a direct link between exposure to EDs and infertility, altered sex ratios at birth, defective embryonic development, low sperm count, altered sperm function, increased morphological abnormalities in spermatozoa, DNA damage, and chromatin remodeling (Auger et al. 2010; Mocarelli et al. 2000; Ryan et al. 2002; Sikka and Wang 2008). These anomalies are caused by ED exposure during gestation, lactation, puberty, and adulthood, or throughout life (Auger et al. 2010; Hayes et al. 2010; Kinch et al. 2015; Phillips and Tanphaichitr 2008). However, despite extensive study, the precise molecular mechanisms underlying how EDs affect fertility are poorly understood.

Bisphenol A (BPA) is a ubiquitous ED used extensively in the production of diverse household products. Exposure to BPA is widespread, both via dietary and nondietary routes. The U.S. Centers for Disease Control and Prevention (CDC) has reported measurable levels of BPA in urine samples of > 90% of the U.S. population (Calafat et al. 2005). BPA affects cellular physiology by binding with diverse physiological receptors, such as genomic and membrane-bound estrogen receptors, androgen receptor, peroxisome receptor γ, and thyroid receptor (Peretz et al. 2014). A review of the literature showed that maternal exposure to BPA was linked to increased incidence of hepatic tumors (Weinhouse et al. 2014), lung inflammation (Bauer et al. 2012), Parkinson disease (Huang et al. 2014), coat color variation (Rosenfeld et al. 2013), and reproductive abnormalities in offspring. Gestational exposure to BPA (< 50 mg/kg bw/day) was reported to decrease the proportion of elongated spermatids in seminiferous tubules in pubertal mice (Okada and Kai 2008), to reduce sperm counts in rats (Salian et al. 2009), and to impair steroidogenesis in rodents (Peretz et al. 2014). More recently, several studies reported that BPA at a dose of < 50 mg/day induced breaks in DNA strands and generated reactive oxygen species (ROS) in spermatozoa (Dobrzyńska and Radzikowska 2013; Liu et al. 2013; Yu et al. 2013). Other studies showed that exposure of two-cell mouse embryos to BPA (100 μM) compromised embryonic development *in vitro* (Takai et al. 2000, 2001). Similarly, we recently reported detrimental, dose-dependent effects of BPA on sperm function, fertilization, and selected fertility-related proteins in spermatozoa (Rahman et al. 2015). Although the results of these studies are consistent, there is insufficient evidence to draw conclusions about how BPA affects sperm function, fertility, and related processes. In addition, these studies did not consider the broader health implications of BPA exposure, which needs further attention.

The combination of two-dimensional gel electrophoresis (2-DE) and electrospray ionization mass spectrometry (ESI-MS/MS) has provided a high-throughput, industrial-scale method for identifying sperm-specific proteins that are indicative of exposure to potentially harmful chemicals (Benninghoff 2007). Proteins associated with a particular chemical exposure can be identified by comparing protein expression patterns between treated and control subjects. Because spermatozoa are known to be largely incapable of transcription and translation (Rahman et al. 2013), they are ideal for proteomic analysis (Kwon et al. 2015a, 2015b). Using an ICR mouse model, we tested the effects of BPA exposure at three different concentrations that are considered to be safe: the tolerable daily intake value (TDI; 50 μg/kg bw/day), the no-observed-adverse-effect-level (NOAEL; 5 mg/kg bw/day), and the lowest-observed-adverse-effect level (LOAEL; 50 mg/kg bw/day) (Bolt and Stewart 2011; Tyl 2009). First, we investigated the effects of gestational BPA exposure on several important characteristics of spermatozoa in adult animals. These included spermatogenesis, sperm count, sperm motility parameters, sperm viability, intracellular ATP, ROS, lactose dehydrogenase (LDH; a measure of cytotoxicity), ionic calcium [Ca^2+^] levels, and capacitation status. We also assessed the fertility (litter size) of adults and investigated the potential mechanisms of action in the subsequent generation. Second, we identified proteins in the spermatozoa that were associated with BPA exposure to determine whether the proteomic alterations could explain the observed functional modifications in spermatozoa. Finally, we used computational bioinformatics tools to evaluate the potential health impacts of BPA exposure based on the patterns of differential protein expression we observed in spermatozoa.

## Methods

### BPA Dose Selection

The exposure scheme consisted of three reference doses of BPA: 50 μg/kg bw/day, 5 mg/kg bw/day, and 50 mg/kg bw/day. The 50 μg/kg bw/day dose represents the TDI value of BPA as defined by the European Food Safety Authority (Bolt and Stewart 2011; Hengstler et al. 2011). The 5 and 50 mg/kg bw/day doses are defined by the U.S. Environmental Protection Agency (EPA) as the NOAEL and LOAEL values for BPA (Hengstler et al. 2011; Tyl 2009). The control animals were treated with corn oil only. An estrogenic positive control was not included in this study because it has been reported that BPA acts in a different way from the estrogenic positive control (diethylstilbestrol) in CD-1 mice (Peretz et al. 2014).

### Experimental Design, Exposure Conditions, and Sample Collection

**Figure 1:**
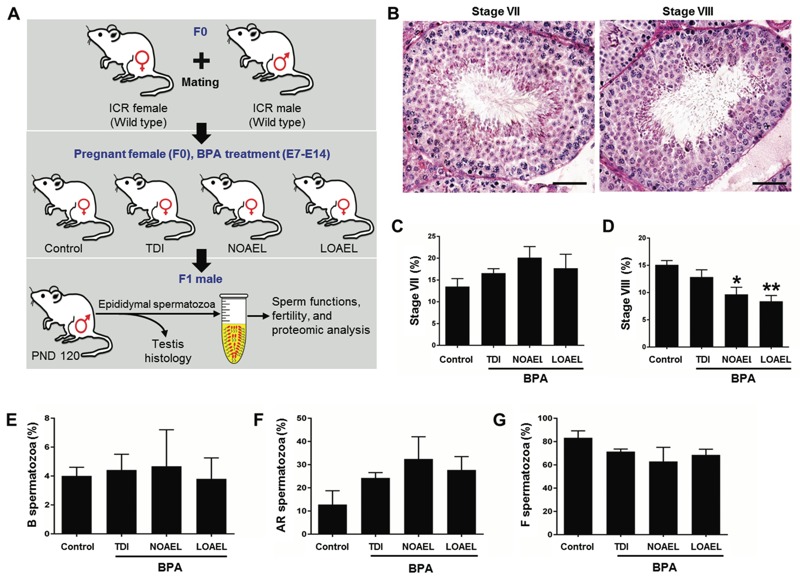
Experimental design (*A*), effects of gestational BPA on testicular seminiferous epithelial cells (*B–D*), and capacitation status (*E–G*) of F1 mice. (*A*) Pregnant mice (F0) were gavaged with BPA at three different doses on embryonic days 7 to 14 (E7 to E14; see text for details). The control mice were treated with corn oil. Testicular histology, sperm count, function tests, and proteomic analyses of spermatozoa from F1 male were conducted on postnatal day (PND) 120. (*B*) Representative photomicrographs showing stage VII and VIII seminiferous epithelial cells (bar = 50 μm). (*C*) Percentage of stage VII seminiferous epithelial cells (mean ± SEM; *n* = 7 mice/group). (*D*) Percentage of stage VIII seminiferous epithelial cells (mean ± SEM; *n* = 7 mice/group). *p < 0.05 and **p < 0.01, compared with control. (*E*) Percentage of live capacitated (B) spermatozoa. (*F*) Percentage of live acrosome-reacted (AR) spermatozoa. (*G*) Percentage of live noncapacitated (F)**spermatozoa. Data of the capacitation status are the means of four replicate experiments ± SEM (*n *= 3 mice/replicate). All data were analyzed using one-way analysis of variance (ANOVA). Tukey’s test was used to identify differences between treatments.

All animal procedures were approved by the Institutional Animal Care and Use Committee of Chung-Ang University, Seoul, Korea. Animals were treated humanely and with regard for alleviation of suffering. The experimental design is depicted in Figure 1A. Sexually mature CD-1 (ICR) mice were bred by cohabitating at the ratio of two females per male in a single cage (see “Animal care and maintenance” in the Supplemental Material for additional details). The females were examined daily and confined individually after observing a vaginal sperm plug [embryonic day 0 (E0)]. The copulated female mice (F0) were randomly assigned into four treatment groups (*n* = 10 mice per group): group 1, control; group 2, TDI; group 3, NOAEL; and group 4, LOAEL. The F0 dams were gavaged daily from E7 to E14 according to specific exposure protocols based on daily changes in body weight. Dosing to the dam was initiated at E7 to avoid the effect of BPA on early embryonic development (E1 to E3) and implantation (E4 to E6). Notably, exposure during the treatment window was critical because primordial germ cell migration, DNA methylation in male and female germ cells, remethylation in male germ cells, sex determination, and epigenomic changes occur between E7 and E14 (Doyle et al. 2013). All copulated females (F0) in different experimental groups were pregnant and gave birth normally. The average litter sizes for the control, TDI, NOAEL, and LOAEL groups were 12.44, 12.17, 11.88, and 11.92 pups, respectively. Following parturition, the offspring (F1) were sexed by examining their anogenital distance. F1 male offspring (4–5 pups/litter) were kept with the F0 lactating mothers in each group until postnatal day 21 (PND 21). All mice (F1) were weaned on PND 22 and then maintained in separate cages until adulthood (PND 120). On PND 120, the adult F1 males from BPA-exposed and control dams were anesthetized and sacrificed to collect the testes and spermatozoa (see “Animal sacrifice and collection of testes and spermatozoa” in the Supplemental Material for additional details).

### Testis Histology, Sperm Function Tests, and Fertility Assessment

See “Histological examination of the testes,” “Sperm count, motility parameters, and viability,” “Assessment of sperm capacitation status,” “Quantitative detection of the intracellular ATP, ROS, Ca2+, and LDH levels,” and “Fertility assessment of F1 males” in the Supplemental Material for the detailed procedures of histological examination of the testes, sperm function tests, and fertility assessment of the F1 male mice.

### Recovery of Spermatozoa for Proteomic Analysis, 2-DE, and Identification of Proteins

The spermatozoa collected from F1 male mice were washed twice by centrifugation (100 × *g* for 2.5 min) at 23 ± 1°C, resuspended in basic medium (BM), and then allowed to swim up for 15 min at 37°C. As previously reported, separation by density gradient centrifugation minimizes contamination (Kwon et al. 2014, 2015b) but results in poor sperm recovery rates by 2-DE (Auger et al. 2010; Cao et al. 2006). We therefore used the swim-up technique to separate the motile sperm fraction from immature spermatozoa and somatic cells. Later, after collection of the motile sperm fraction, the spermatozoa were carefully checked for the absence of immature spermatozoa and somatic cells. To avoid the individual-dam effect, each set of spermatozoa was collected randomly and mixed together for proteomic analysis. The details of the 2-DE and protein identification procedures are described elsewhere (Kwon et al. 2015a, 2015b) (see “2-DE and identification of proteins” in the Supplemental Material for additional details).

### Western Blots

We performed Western blotting to detect phospho-PKA substrates and tyrosine phosphorylation and to confirm the 2-DE results (see “Western blots” in the Supplemental Material for additional details of the Western blotting procedure).

### Signaling Pathways

The Pathway Studio program (Elsevier, Amsterdam, Netherlands) was used to predict the signaling pathways and the clinical significance of proteins differentially expressed under the BPA treatments according to previously described methods (Kwon et al. 2014, 2015b).

### Statistical Analysis

Results from three or more independent experiments are expressed as the mean ± standard error of the mean (SEM). We used SPSS (v. 18.0; SPSS Inc.) for all statistical analyses; we performed multiple comparisons with Tukey’s multiple comparison test. The probabilities of the signaling pathways of differentially expressed proteins were determined using Fisher’s exact test. Statistical significance was assumed at *p* < 0.05 in all instances.

## Results

### Gestational Exposure to BPA Affects Stage Distribution of the Seminiferous Epithelium

First, we evaluated whether gestational exposure to BPA affected spermatogenesis in adulthood. The relative frequency of stage VII and VIII seminiferous epithelial cells in testis was evaluated. Significant decreases in stage VIII were observed following BPA exposure at NOAEL and LOAEL doses (Figure 1D). However, the percentage of stage VII seminiferous epithelial cells in testis appeared to be unaffected (Figure 1C).

### Gestational Exposure to BPA Affects Motility, Motion Kinematics, and Intracellular ATP Levels in Adult Mouse Spermatozoa

**Figure 2:**
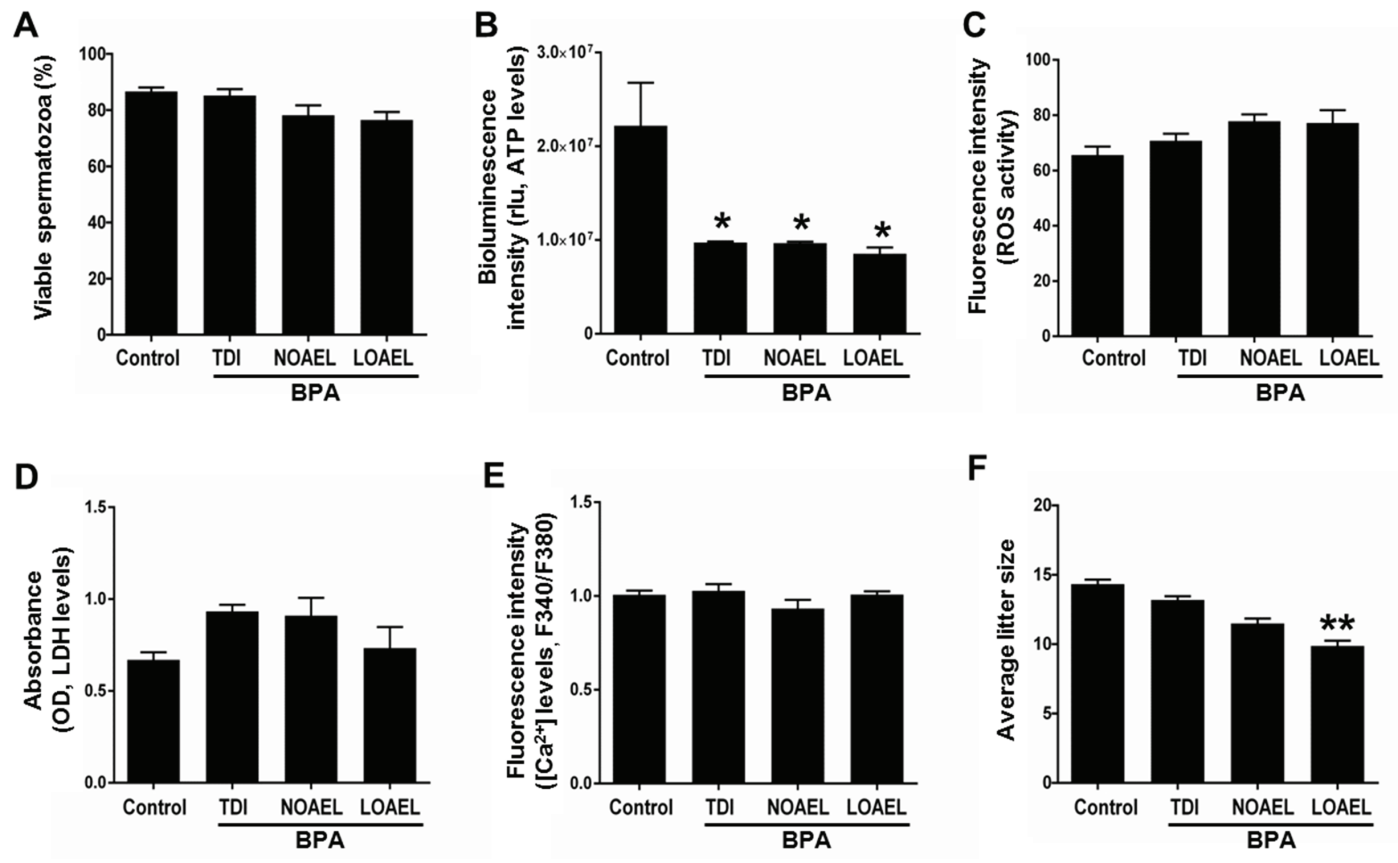
Effects of gestational bisphenol-A (BPA) exposure on several sperm parameters (*A–E*) and litter size of F1 male mice. (*A*) Percentage of viable spermatozoa. (*B*) Bioluminescence intensity (proportional to the levels of intracellular ATP). (*C*) Fluorescence intensity [proportional to the intracellular reactive oxygen species (ROS) activity]. (*D*) Absorbance [proportional to the levels of intracellular lactose dehydrogenase (LDH)]. (*E*) Fluorescence intensity [proportional to the levels of intracellular ionic calcium (Ca^2+^)]. (*F*) Average litter size by F1 male mice. *p < 0.05, compared with control. **p < 0.01, compared with control. Data are the means of four replicate experiments ± SEM (*n *= 3 mice/replicate). All data were analyzed using one-way analysis of variance (ANOVA). Tukey’s test was used to identify differences between treatments.

Next, we evaluated whether gestational exposure to BPA affected the function and biochemical properties of spermatozoa in adult mice. Sperm count (concentration), linearity (LIN), and wobble (WOB) were significantly affected by both NOAEL and LOAEL doses of BPA. However, percentage of motility (MOT) was only affected by the LOAEL dose (*p* < 0.01; Table 1). Although all doses of BPA dramatically decreased intracellular ATP levels (*p* < 0.05), other parameters—including viability, levels of intracellular ROS, LDH, Ca^2+^, and capacitation status—were apparently unaffected (Figures 1 and 2).

**Table 1:** Adult (F1) sperm count and motility parameters after gestational BPA exposures.

Parameters	Control^*a*^	TDI^*b*^	NOAEL^*c*^	LOAEL^*d*^
Concentration (10^6^/mL)	77.33 ± 4.37	70.73 ± 2.72	54.64 ± 1.85**	48.43 ± 3.17**
MOT (%)	83.35 ± 0.93	77.01 ± 1.11	79.69 ± 2.46	71.82 ± 0.63**^, #^
HYP (%)	33.62 ± 0.03	33.06 ± 1.97	32.83 ± 3.15	31.85 ± 0.73
VCL (μm/sec)	192.22 ± 3.64	178.96 ± 4.69	183.06 ± 5.69	175.55 ± 1.67
VSL (μm/sec)	93.7 ± 0.58	80.59 ± 2.56	89.00 ± 4.52	67.77 ± 1.05
VAP (μm/sec)	94.37 ± 1.85	84.16 ± 2.54	89.99 ± 1.42	76.55 ± 3.98
ALH (μm)	7.736 ± 0.78	7.17 ± 1.36	7.34 ± 0.36	6.93 ± 0.78
LIN (%)	59.66 ± 1.25	53.56 ± 1.25	46.98 ± 0.58*	47.46 ± 0.78*
WOB (%)	61.89 ± 1.23	56.90 ± 0.56	54.90 ± 0.54*	52.63 ± 0.59*
Notes: ALH, mean amplitude of head lateral displacement; BPA, bisphenol A; Concentration, sperm count; HYP, hyperactivated motility; LIN, linearity; LOAEL, lowest observed adverse effect level; MOT, motility; NOAEL, no observed adverse effect level; VAP, average path velocity; VCL, curvilinear velocity; VSL, straight-line velocity; WOB, wobble. Data are presented as the mean ± SEM. Data are the means of three replicate experiments ± SEM (*n* = 3 mice/replicate). All data were analyzed by one-way analysis of variance (ANOVA) with multiple comparisons and Tukey’s multiple comparison test. Concentration, **p < 0.01, compared with control. MOT, **p < 0.01, compared with control; and ^#^*p* < 0.05 for LOAEL compared with NOAEL. LIN, *p < 0.05 compared with control and TDI. WOB, *p < 0.05 compared with control and TDI. ^***a***^Corn oil. ^***b***^TDI = 50 μg/kg bw/day. ^***c***^NOAEL = 5 mg/kg bw/day. ^***d***^LOAEL = 50 mg/kg bw/day.				

### Mechanistic Evolution of BPA-Induced Alteration of Sperm Function

**Figure 3:**
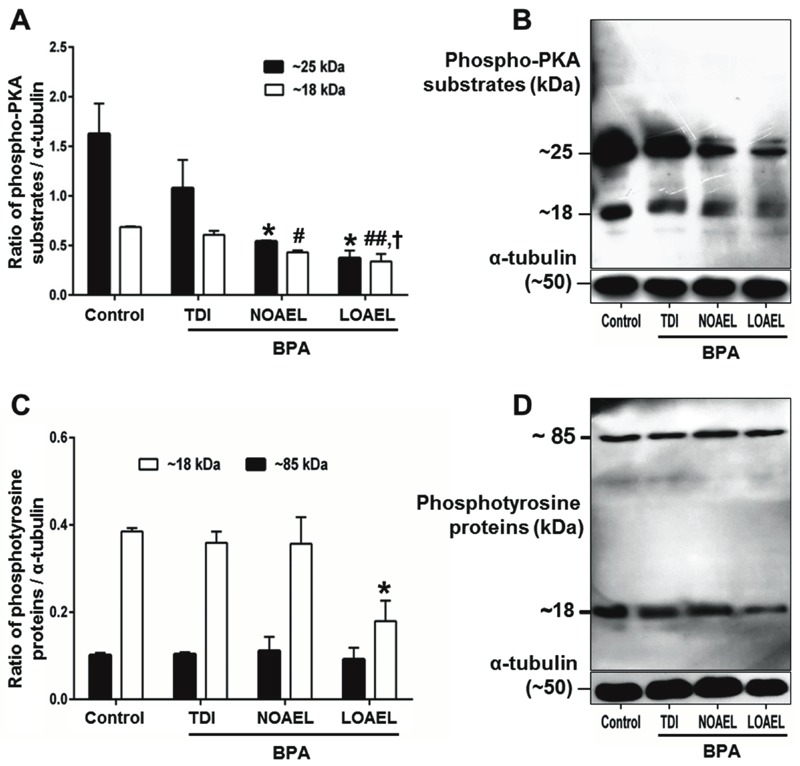
PKA activity (*A–B*) and tyrosine phosphorylation levels (*C–D*) in spermatozoa. (*A*) Density of phospho-PKA substrates in ~ 25 kDa (*p < 0.05, compared with control) and ~18 kDa [^#^ *p* < 0.05 and ^##^ *p* < 0.01, compared with control; ^†^ *p* < 0.05 for lowest observed adverse effect level (LOAEL) compared with tolerable daily intake value (TDI)]. (*B*) Representative Western blot image of phospho-PKA substrates. (*C*) Density of phosphotyrosine proteins in ~18 kDa (*p < 0.05, compared with control) and ~85 kDa. (*D*) Representative Western blot image of phosphotyrosine proteins. Data are the means of four replicate experiments ± SEM (*n *= 3 mice/replicate). All data were analyzed using one-way analysis of variance (ANOVA). Tukey’s test was used to identify differences between treatments.

PKA activity and protein tyrosine phosphorylation are critical during functional modifications in mammalian spermatozoa that are required for fertilization (Rahman et al. 2014, 2015). Therefore, we next tested the hypothesis that the functional modifications in spermatozoa are regulated via PKA activity and protein phosphorylation status at tyrosine residues. Our results showed that both NOAEL and LOAEL doses significantly reduced PKA activity (to 25 and 18 kDa), and LOAEL doses of BPA significantly reduced tyrosine phosphorylation levels (to 18 kDa) in spermatozoa (Figure 3).

### Gestational Exposure to BPA Affects Average Litter Size of F1 Male Mice

The average litter sizes for the treatments (TDI, 13.2; NOAEL, 11.5; and LOAEL, 9.8 pups) were smaller than that of the control group (14.33 pups). However, only the LOAEL treatment was significantly different when all of the groups were compared together (*p* < 0.01; Figure 2F).

### Differentially Expressed Proteins in Control and BPA-Exposed Spermatozoa

On average, 284 spots were consistently detected in all gels, 250 of which shared similar expression patterns across treatments. Twenty-five spots showed a dose-dependent expression profile, but significant (*p <* 0.05) changes were observed in only 8 spots. Six of these spots were identified by ESI-MS/MS (Table 2; see Figure S1). In our assessment of the dose-dependent expression profiles, we avoided 9 spots that demonstrated nonlinear differences in expression between treated and control groups. Among the identified proteins, we observed decreases in the expression of phospholipid hydroperoxide glutathione peroxidase (PHGPX); ATP synthase subunit O, mitochondrial (ATP5O); glutathione *S*-transferase Mu 5 (GSTM5), mitochondrial NADH dehydrogenase [ubiquinone] 1 alpha subcomplex subunit 10 (NDUFA10). By contrast, we observed increases in isoaspartyl peptidase/L-asparaginase (ASRGL1) and mitochondrial superoxide dismutase [Mn] (SOD2). ATP5O, GSTM5, ASRGL1, and SOD2 levels were significantly different in all treated groups compared with the control. Although PHGPX levels were significantly altered by both the NOAEL and LOAEL doses, we observed noticeable changes in NDUFA10 under the LOAEL treatment (Table 2).

**Table 2:** List of differentially expressed proteins in adult mouse spermatozoa following gestational exposure to BPA.

Protein	Symbol	gi Number	Peptide sequence	Matched peptide	Sequence coverage (%)	MASCOT score^*a*^	Relative intensity			
							Control^*b*^	TDI^*c*^	NOAEL^*d*^	LOAEL^*e*^
Phospholipid hydroperoxide glutathione peroxidase	PHGPX	gi|2522259	R.YGPMEEPQVIEK.D	710.35	6.09	66	1.00	0.53 ± 0.18	0.43 ± 0.04*	0.40 ± 0.08*
ATP synthase subunit O, mitochondrial	ATP5O	gi|20070412	K.VSLAVLNPYIK.R	608.87	5.16	52	1.00	0.34 ± 0.06****	0.40 ± 0.01****	0.45 ± 0.07***
Glutathione *S*-transferase Mu	GSTM5	gi|6754086	K.LTFVDFLTYDVLDQN.R	980	7	45	1.00	0.35 ± 0.08**	0.50 ± 0.12**	0.43 ± 0.07**
NADH dehydrogenase [ubiquinone] 1 alpha subcomplex subunit 10, mitochondrial	NDUFA10	gi|13195624	R.LQSWLYASR.L	562.3	10.11	33.3	1.00	0.50 ± 0.19	0.51 ± 0.17	0.20 ± 0.04*
Isoaspartyl peptidase/ʟ‑asparaginase	ASRGL1	gi|81875980	K.FAEDMGIPQVPVEK.L	780.4	4.29	65	1.00	3.99 ± 0.17*	4.14 ± 0.72*	3.36 ± 0.43*
Superoxide dismutase [Mn], mitochondrial	SOD2	gi|31980762	K.GDVTTQVALQPALK.F	720.39	6.3	55	1.00	2.82 ± 0.40**	2.79 ± 0.39*	2.80 ± 0.13**
Notes: BPA, bisphenol A; gi Number, GenInfo Identifier; LOAEL, lowest observed adverse effect level; NOAEL, no observed adverse effect level; TDI, tolerable daily intake value. Relative spot intensity of PHGPX (*p < 0.05, compared with control), ATP5O (***p < 0.001 and ****p < 0.0001, compared with control), GSTM5 (**p < 0.01, compared with control), NDUFA10 (*p < 0.05, compared with control), ASRGL1 (*p < 0.05, compared with control), and SOD2 (*p < 0.05 and **p < 0.01, compared with control) are presented as the mean ± SEM (*n* = 3 mice/replicate). Data were analyzed by one-way analysis of variance (ANOVA) with multiple comparisons, with Tukey’s multiple comparison test. ^***a***^MASCOT score is –log_10_(*p*), where *p* is the probability that the observed match is a random event. Individual scores > 30 indicate identity or extensive homology (*p* < 0.05). ^***b***^Corn oil. ^***c***^TDI = 50 μg/kg bw/day. ^***d***^NOAEL = 5 mg/kg bw/day. ^***e***^LOAEL = 50 mg/kg bw/day.										

### Validation of 2-DE Findings by Western Blot Analysis

**Figure 4:**
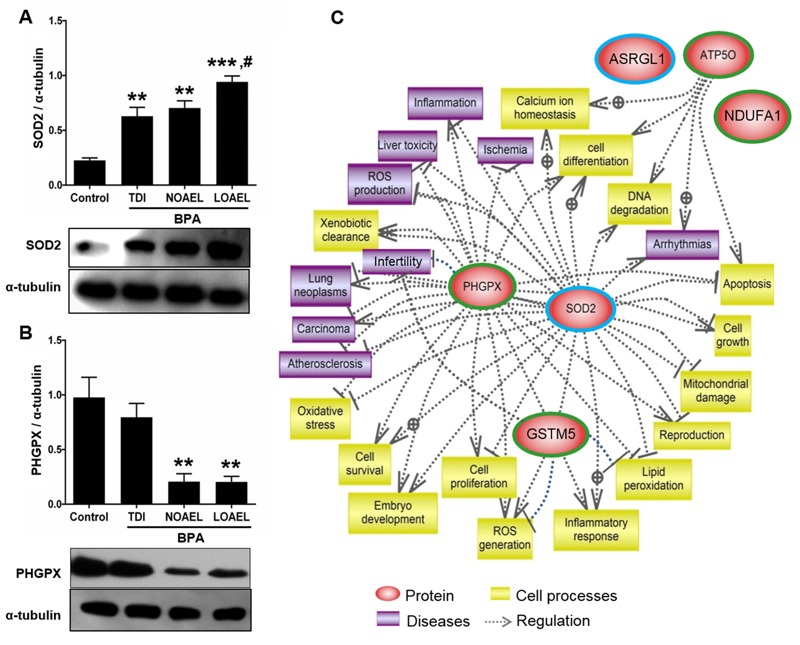
Western blot analysis (*A–B*) and differentially expressed proteins regulating cellular processes and diseases. (*A*) Densities of measured SOD2 [**p < 0.01 and ***p < 0.001, compared with control; ^#^ *p* < 0.05 for lowest observed adverse effect level (LOAEL) compared with tolerable daily intake value (TDI)]. (*B*) Densities of PHGPX (**p < 0.01, compared with control). Data are the means of three replicate experiments ± SEM (*n *= 3 mice/replicate). All data were analyzed using one-way analysis of variance (ANOVA). Tukey’s test was used to identify differences between treatments. (*C*) Regulatory cellular process and possible health hazards of the differentially expressed proteins identified in spermatozoa. Proteins with significantly (*p* < 0.05) higher or lower expression levels are indicated by blue and green circles, respectively.

Levels of SOD2 and PHGPX were further evaluated by Western blotting to validate the 2-DE findings. We detected SOD2 and PHGPX in spermatozoa at ~25 and 22 kDa, respectively. The expression profiles of both proteins in the Western blots corresponded to their values as measured with 2-DE (Figure 4A,B).

### Signaling Pathways

Next, we searched gene ontology annotations in a bioinformatics database for signaling pathways featuring the differentially expressed proteins we identified. We found four canonical pathways (*p* < 0.05; see Table S1). Of the proteins we identified, ATP5O, PHGPX, SOD2, and GSTM5 showed significant connections to several diseases, including neoplasia, carcinoma, infertility, and others known to be related to oxidative damage, lipid peroxidation, and apoptosis (Figure 4C).

## Discussion

BPA is a ubiquitous ED with widespread industrial applications in the manufacturing of household products. The basic mechanisms by which BPA affects reproduction are reasonably well-studied, and it is known to possess antiandrogenic and/or antiestrogenic actions, to manipulate the hypothalamic–pituitary–gonadal hormone feedback system, and to prompt germ cell apoptosis, thereby impairing spermatogenesis (Jin et al. 2013; Lassen et al. 2014). However, the functional and biochemical changes in spermatozoa caused by BPA exposure and their underlying mechanisms remain elusive. Given the toxicological significance of BPA, we investigated whether gestational exposure to BPA (at TDI, NOAEL, and LOAEL doses) could affect sperm function and fertility in adulthood. We also elucidated the protein expression profiles in spermatozoa following BPA exposure to assess whether alterations in the proteome could be responsible for these functional modifications.

We found that gestational exposure to BPA (NOAEL and LOAEL) significantly decreased the number of stage VIII seminiferous epithelial cells in testis of F1 mice (Figure 1D). BPA has been shown to induce testicular germ cell apoptosis, specifically at stages VII and VIII (D’Souza et al. 2005). Treatment with BPA (200 μg/kg bw/day) for 60 days has been shown to increase stage VII and decrease stage VIII seminiferous epithelium in adult rats (Liu et al. 2013, 2014). We did not find any significant changes in the number of stage VII seminiferous epithelial cells (Figure 1C); however, the observed reduction in the frequency of stage VIII seminiferous epithelial cells suggests a possible delay in the spermiation process that occurs at stage VIII (Liu et al. 2014). Although the reasons for these inconsistencies in the frequency of stage VII seminiferous epithelium are unclear, direct exposure to BPA for a long time (60 days) may have induced changes that were different from those observed in F1 males that received gestational exposure. Further, a decrease in the number of stage VIII seminiferous epithelial cells might directly correlate with a smaller sperm count, as was noted in the present study. BPA exposure also caused reductions in several parameters (e.g., motility, LIN, and WOB), with significant decreases at higher doses (Table 1). Thus, gestational exposure to BPA might adversely affect spermatogenesis in adulthood, predisposing individuals to have small numbers of sperm with decreased motility. Our findings are consistent with those reported by other investigators (Liu et al. 2013, 2014).

Measuring the percentage of motile spermatozoa is likely the most straightforward approach to evaluating male fertility because immotile sperm cannot fertilize an oocyte (Rahman et al. 2014; Suarez 2008). In addition, individuals with a sub-optimal quantity or quality of spermatozoa are less likely to fertilize an oocyte (Suarez 2008). To investigate how gestational BPA exposure affects sperm motility, we evaluated several biochemical parameters in spermatozoa that collectively regulate motility, including viability, intracellular ROS, Ca^2+^, ATP and LDH levels, PKA activity, and protein tyrosine phosphorylation (Rahman et al. 2014, 2015; Suarez 2008). We did not find any changes in viability, intracellular ROS, LDH, or Ca^2+^ levels. However, intracellular ATP levels, PKA activity, and protein tyrosine phosphorylation were strikingly decreased in spermatozoa following BPA exposure (Figures 2 and 3). It has been demonstrated that phosphorylation of mitochondrial proteins regulates mitochondrial biogenesis to produce the ATP that fuels sperm motility (Miki et al. 2004). In addition, several studies indicate that sperm motility is regulated by cAMP-dependent and PKA-mediated phosphorylation of sperm proteins, mainly on tyrosine residues (Naz and Rajesh 2004; Turner et al. 1999; Visconti 2009). Therefore, it is tempting to hypothesize that gestational exposure to BPA down-regulates PKA activity and protein tyrosine phosphorylation, leading to decreased ATP production and affecting sperm motility and fertility. Decreased ATP levels in spermatozoa were also reported in another study after 6 hr of *in vitro* exposure to BPA, but PKA activity and tyrosine phosphorylation were increased (Rahman et al. 2015). Although the reasons for these inconsistencies are unknown, direct *in vitro* exposure to BPA for 6 hr may have induced capacitation-related changes that were different from those induced *in vivo*.

Here, we provide initial evidence demonstrating that gestational exposure to BPA alters sperm protein profiles in adults of the subsequent generation. However, it is important to assess the mechanisms underlying the functional modifications observed in spermatozoa. Six proteins showed significant dose-dependent differences in expression profiles when the treatment groups were compared with the control (Table 2; see Figure S1). PHGPX and GSTM5 are stress response proteins predominantly expressed in the sperm midpiece mitochondria and in the fibrous sheath, respectively (Ijiri et al. 2014; Imai et al. 2001). Both proteins play critical roles in the suppression of mitochondrial ROS generation by scavenging H_2_O_2_ and preventing oxidative injury (Nomura et al. 1999). As a selenoprotein, PHGPX regulates mitochondrial ATP production to maintain proper sperm motility. GSTM5 also plays a critical role in the regulation of sperm motility (Inaba 2011). In the present study, the density of both proteins decreased in spermatozoa after gestational exposure to BPA (Table 2), suggesting that BPA down-regulated the expression of both proteins and thus affected sperm function and fertility. Consistent with these findings, Imai et al. (2001) reported that spermatozoa from oligoasthenozoospermic infertile men were unable to express mitochondrial PHGPX.

ATP5O plays an important role in cellular metabolism and oxidative phosphorylation. Ijiri et al. (2011) identified ATP5O as a cauda epididymal sperm-differential protein responsible for ATP biogenesis. In the present study, decreased expression of this protein might have a direct correlation with the reduced ATP levels and motility observed after BPA exposure. In contrast, NDUFA10 is an NADH dehydrogenase (ubiquinone) subunit that plays a potential role in the electron transport chain (Emahazion et al. 1998). An extensive literature search revealed that the functions of this protein in spermatozoa have not been studied. Because this protein is an enzyme in the electron transfer chain, its down-regulation might directly affect ATP production. ASRGL1 is another protein that is up-regulated in spermatozoa after gestational BPA exposure. Increased expression of this protein has been reported in the testis (Bush et al. 2002), but its function in spermatozoa remains unknown. Further studies are needed to elucidate the function of these proteins in spermatozoa as well as their relationship to BPA exposure. SOD2 plays a significant role in the antioxidant defense system in spermatozoa and protects against oxidative damage (Yan et al. 2014). Yu et al. (2013) reported decreased superoxide dismutase activity in the semen of heavy smokers, predisposing them to reduced fertility. We observed that gestational exposure to all BPA doses significantly increased SOD2 expression in spermatozoa (Table 2). It is therefore tempting to hypothesize that BPA affects SOD2 activity even at very low doses (TDI) and that it may be correlated with the fertility of the exposed individual.

We showed that five out of the six identified proteins (PHGPX, GSTM5, SOD2, NDUFA10, and ATP5O) were associated with four canonical pathways (see Table S1). Because the interaction of these pathways is critical for cellular physiology, alterations of their constituent protein profiles could be directly responsible for pathological outcomes. We found that four proteins (PHGPX, GSTM5, SOD2, and ATP5O) were potentially linked to infertility, neoplasia, carcinoma, and other disorders, perhaps as a consequence of oxidative stress, lipid peroxidation, apoptosis, cell proliferation, ionic homeostasis, or a combination of these processes (Figure 4C). Thus, adult males might have increased susceptibility to these pathologies after gestational BPA exposure. However, further studies are required to validate our preliminary findings and to better evaluate the possible clinical implications.

## Conclusions

To the best of our knowledge, this is the first comprehensive *in vivo* study to evaluate the effects of gestational BPA exposure on adult F1 sperm function, fertility, and related processes. BPA at the TDI dose had little to no effect on spermatozoa, but the NOAEL and LOAEL doses had severe, toxic effects on spermatogenesis and on several sperm parameters. These doses also induced changes in the sperm proteome that could affect the fertility of the exposed individual. Therefore, it is of critical public health importance to reevaluate the levels of BPA exposure that are currently deemed to be acceptable.

## Supplemental Material

(2.1 MB) PDF
