# Supplementary material for: Gestational Exposure to Bisphenol A Affects the Function and Proteome Profile of F1 Spermatozoa in Adult Mice
Source: Environ Health Perspect. 2016 Jul 6;125(2):238–45. doi: 10.1289/EHP378 (PMC5289913; doi:10.1289/EHP378)
Supplement: (2.1 MB) PDF [file EHP378.s001.acco.pdf]

Environ Health Perspect
DOI: 10.1289/EHP378

Note to readers with disabilities: *EHP* strives to ensure that all journal content is accessible to all readers. However, some figures and Supplemental Material published in *EHP* articles may not conform to 508 standards due to the complexity of the information being presented. If you need assistance accessing journal content, please contact ehp508@niehs.nih.gov. Our staff will work with you to assess and meet your accessibility needs within 3 working days.

Supplemental Material

Gestational Exposure to Bisphenol-A Affects the Function and Proteome Profile of F1 Spermatozoa in Adult Mice

Md Saidur Rahman, Woo-Sung Kwon, Polash Chandra Karmakar, Sung-Jae Yoon, Buom-Yong Ryu, and Myung-Geol Pang

Table of Contents

Chemicals, reagents, and media
Animal care and maintenance
Animal sacrifice and collection of testes and spermatozoa
Histological examination of the testes
Sperm count, motility parameters, and viability
Assessment of sperm capacitation status
Quantitative detection of the intracellular ATP, ROS, Ca^{2+}, and LDH levels
Fertility assessment of F1 males
2-DE and identification of proteins
Western blots
Validation Compliance Checker
References
Table S1. Signaling pathways significantly ($P < 0.05$) associated with differentially expressed proteins from F1 spermatozoa
Figure S1. 2-DE protein spot patterns from adult spermatozoa. (A) Control (corn oil). (B) TDI treatment. (C) NOAEL treatment. (D) LOAEL treatment

Supplemental Text

Chemicals, reagents, and media. All chemicals and reagents were purchased from Sigma-Aldrich, unless otherwise mentioned. BPA (≥99% pure) was dissolved directly in corn oil to obtain the desired concentration. Modified Tyrode's medium (97.84 mM NaCl, 1.42 mM KCl, 0.47 mM MgCl2·H2O, 0.36 mM NaH2PO4·H2O, 5.56 mM D-glucose, 25 mM NaHCO3, 1.78 mM CaCl2·H2O, 24.9 mM Na-lactate, 0.47 mM Na-pyruvate and 50 μg/mL gentamycin; osmolality 300 ± 20 mOsm/kg; pH 7.2 ± 0.2) was freshly prepared and used as a basic medium (BM) for the spermatozoa (Rahman et al. 2014). The BM was supplemented with bovine serum albumin (4 mg/ml) and was incubated overnight prior to the day of experiment at 37 °C under 5% CO_2.

Animal care and maintenance. Eight-week old CD-1 (ICR) outbred male and female mice (F0) were purchased from Doo Yeol Biotech, Seoul, Korea. The mice were housed individually in prewashed polysulfone cages with autoclaved bedding materials. All animals were kept under constant conditions of temperature (22 ± 2 °C), humidity, ventilation, and light (12-h light/dark period) for four weeks before mating. All mice were provided ad libitum access to a soy-free diet (SAM#31; Samtako Co.) and to water from glass bottles throughout the study period.

Animal sacrifice and collection of testes and spermatozoa. Using sterilized surgical forceps and scissors, the testes were carefully collected from each mouse (F1) and then fixed in 10% neutral buffered formalin for 24 h for histological examination. Simultaneously, spermatozoa from the caput and cauda epididymides were collected and the surrounding fat was removed. These samples were then placed into sterile cell culture dishes containing BM supplemented with 0.4%

BSA. Subsequently, the caput or cauda epididymides were punctured with a needle to release the spermatozoa from the ducts. To achieve proper dispersion, the spermatozoa from the cauda epididymides were further incubated at 37 °C in 5% CO_2 for 10 min.

Histological examination of the testes. One testis from each mouse (n = 7 mice per group) was embedded in paraffin, sectioned at 5 μm, and stained with PAS-H for histological analysis. The sections were analyzed using a light microscope (Eclipse, Nikon) with a 40×objective. PAS-H staining and the staging of the mouse seminiferous tubules were performed as previously described (Ahmed and de Rooij 2009; Liu et al. 2013). We make a preponderance decision method to identify the frequency of the stage VII and VIII. Finally, the results were expressed as the frequency (%) of each stage in the total seminiferous tubules and more than 200 seminiferous tubules per mouse were examined.

Sperm count, motility parameters, and viability. Sperm count and motility parameters were evaluated using CASA (SAIS plus version 10.1; Medical Supply) (Rahman et al. 2014). The program settings were: frames acquired, 20; frame rate, 30 Hz; minimum contrast, 7; minimum size, 5; low/high size gates, 0.4–1.5; low/high intensity gates, 0.4–1.5; non-motile head size, 16; non-motile brightness, 14. We evaluated spermatozoa movement in five randomly selected fields for each replicate. To evaluate sperm viability / membrane integrity, a hypo-osmotic swelling test (HOST) was performed as previously described (Yoon et al. 2015). Finally the spermatozoa were classified broadly as viable or nonviable according to the World Health Organization 2010 manual.

Assessment of sperm capacitation status. Spermatozoa capacitation status was evaluated with combined hoechst 33258/chlortetracycline fluorescence according to a protocol previously used on mice spermatozoa (Rahman et al. 2014). Spermatozoa were evaluated with a Nikon microphot-FXA under epifluorescence illumination. Finally, the spermatozoa were categorized as live capacitated (B, green fluorescence over the acrosomal region and a dark postacrosome), live acrosome reacted (AR, mottled green or no fluorescence fluorescence over the head), or live noncapacitated (F, bright green fluorescence distributed uniformly over the sperm head) according to the criteria of Maxwell and Johnson (Maxwell and Johnson 1999). Three slides per sample with at least 400 spermatozoa per slide were evaluated.

Quantitative detection of the intracellular ATP, ROS, Ca^{2+}, and LDH levels. Unless otherwise noted, we followed existing protocols to measure the intracellular levels of key compounds qualitatively (Rahman et al. 2014). Briefly, intracellular ATP levels were monitored using a commercially available ATP bioluminescence assay kit (CLS II; Roche Molecular Biochemicals). ATP bioluminescence intensity (RLU) was detected using a Microplate Multimode Reader (Promega). As such, the RLU value is proportional to the levels of intracellular ATP levels. Cellular ROS activity were monitored with an oxidation-sensitive fluorescent dye, 2′,7′-dichlorofluorescin diacetate (Abcam). Fluorescence intensity of 2′,7′-dichlorofluorescin was detected by Microplate Fluorometer (GEMINI EM; Molecular Devices) with maximum excitation and emission spectra of 495 and 529nm, respectively. Intracellular Ca^{2+} was measured using 5μM fura-2 acetoxymethyl ester (Molecular Probes). Data were express as the ratio of the fluorescence at 340 to that of 380 nm (F340/F380). SoftMax Pro 5 software (Molecular Devices) was used for the calculations of the intracellular levels of the ROS

and Ca^{2+}. As such, the fluorescence intensity is proportional to the levels of intracellular ROS and Ca^{2+}. On the other hand, LDH levels were assayed using a cytotox 96® non-radioactive cytotoxicity kit (Promega). LDH activity was measured as the absorbance (OD) at 490 nm using a luminometer (Gemini Em) and calculated using SoftMax Pro 5 software. The OD value is comparative to the intracellular levels of LDH.

Fertility assessment of F1 males. On PND 120, five F1 males per group were randomly selected for mating with sexually-mature, untreated CD1 (ICR) females. Each male was housed with three unexposed females. The females were carefully checked for vaginal plugs and then separated into individual cages until parturition. The average litter size was calculated as the total litter size for the group divided by the number of mated females.

2-DE and identification of proteins. The details of the 2-DE and protein identification procedures are described elsewhere (Kwon et al. 2015). Briefly, swim-up sperm fractions (50 $\times 10^6$ /ml) were incubated in the rehydration buffer for protein extraction. First-dimension electrophoresis was performed using 24-cm NL Immobiline DryStrips (pH 3–11; Amersham Biosciences). After subsequent equilibration of the strip, second-dimension electrophoresis was conducted with 12.5% (w/v) SDS-PAGE gels. The 2-DE gel was silver-stained for protein visualization according to manufacturer instructions (Amersham Biosciences). We used silver-staining because it allows efficient detection of in-gel protein variants and is compatible with downstream data processing. A high-resolution GS-800 calibrated densitometer (Bio-Rad Laboratories) was used to identify and compare spots matched between treated and control samples. The excised gel spots were subjected to in-gel trypsin digestion for the detection of

protein as described previously (Kwon et al. 2014; Kwon et al. 2015). Peptides were generated by in-gel digestion and analyzed by mass spectrometry (MS) using tandem nano-electrospray ionization (ESI) on a MicroQ-TOF III mass spectrometer (Bruker Daltonics) at RT. The ESI-MS/MS data were analyzed using a peptide sequence system. MASCOT software (Matrix Science) was used to search for MS/MS ions. Peptide fragments were attained from peptide peaks in ESI-MS by ESI-MS/MS. These fragments were then identified using the Mascot search engine (Matrix Science). The results were limited to *Mus musculus*.

Western blots. Western blots were performed to detect phospho-PKA substrates and tyrosine phosphorylation, and to confirm 2-DE results according to previously described methods (Rahman et al. 2014). Briefly, the pooled samples were washed twice by centrifugation and resuspended in sample buffer containing 5% 2-mercaptoethanol. Samples were then transferred to polyvinylidene fluoride membranes (Amersham Biosciences) for SDS–polyacrylamide gel electrophoresis. Membranes were blocked with 3% blocking agent (Amersham Biosciences) for 1 h at RT. They were then treated with the target antibody suspended in blocking solution. The following substrates were detected: phospho-PKA substrate (Anti-phospho-PKA substrate, rabbit monoclonal antibody [1:10000]; Cell Signaling Technology), phospho-tyrosine (anti-phosphotyrosine antibody [PY20, 1:2500]; Abcam), superoxide dismutase (Anti-SOD2 rabbit polyclonal antibody [1:5000], Abcam) and phospholipid hydroperoxide glutathione peroxidase (Anti-PHGPX Rabbit monoclonal antibody [1:5000], Abcam). To detect proteins on the membrane, we used an enhanced chemiluminescence (ECL) technique. Finally, protein bands were scanned on a GS-800 calibrated imaging densitometer and analyzed with the Quantity One

(Bio-Rad) program. Data were presented as the ratio of the target protein to α-tubulin in treated and control samples.

Validation Compliance Checker.

Search Parameters and Acceptance Criteria (MS/MS and/or PMF data)

Name of peaklist-generating software and release version (number)

DataAnalysis (4.0)

Name of the search engine and release version (number)

Mascot (2.4),

Homology search: sequence similarity searching tool

FASTA (www.ebi.ac.uk/Tools/sss/fasta)

Name of sequence database/spectral library searched and release version/date

Mascot search DB: NCBInr (May 2014)

Homology search DB: UniprotKB/TrEMBL, UniprotKB/Swissprot(Oct 2014)

References

Ahmed EA, de Rooij DG. 2009. Staging of mouse seminiferous tubule cross-sections. Methods Mol Biol 558:263-277.

Kwon WS, Rahman MS, Lee JS, Kim J, Yoon SJ, Park YJ, et al. 2014. A comprehensive proteomic approach to identifying capacitation related proteins in boar spermatozoa. BMC Genomics 15:897.

Kwon WS, Rahman MS, Lee JS, Yoon SJ, Park YJ, Pang MG. 2015. Discovery of predictive biomarkers for litter size in boar spermatozoa. Mol Cell Proteomics 14:1230-1240.

Liu C, Duan W, Li R, Xu S, Zhang L, Chen C, et al. 2013. Exposure to bisphenol a disrupts meiotic progression during spermatogenesis in adult rats through estrogen-like activity. Cell Death Dis 4:e676.

Maxwell WM, Johnson LA. 1999. Physiology of spermatozoa at high dilution rates: The influence of seminal plasma. Theriogenology 52:1353-1362.

Rahman MS, Kwon WS, Lee JS, Kim J, Yoon SJ, Park YJ, et al. 2014. Sodium nitroprusside suppresses male fertility in vitro. Andrology 2:899-909.

Yoon SJ, Kwon WS, Rahman MS, Lee JS, Pang MG. 2015. A novel approach to identifying physical markers of cryo-damage in bull spermatozoa. PLoS One 10:e0126232.

Table S1. Signaling pathways significantly ($P < 0.05$) associated with differentially expressed proteins from F1 spermatozoa.

Name	Overlapping entities	*P*-value	Data Source
Glutathione metabolism	PHGPX, GSTM5	0.021	Ariadne metabolic pathways
B cell activation	SOD2, PHGPX	0.019	Ariadne cell signaling pathways
Respiratory chain and oxidative phosphorylation	NDUFA10, ATP5O	0.013	Ariadne metabolic pathways
ROS metabolism	SOD2, PHGPX	0.015	Ariadne metabolic pathways

BPA-induced, differentially expressed proteins were entered into the Pathway Studio program to identify associated ($P < 0.05$) signaling pathways.

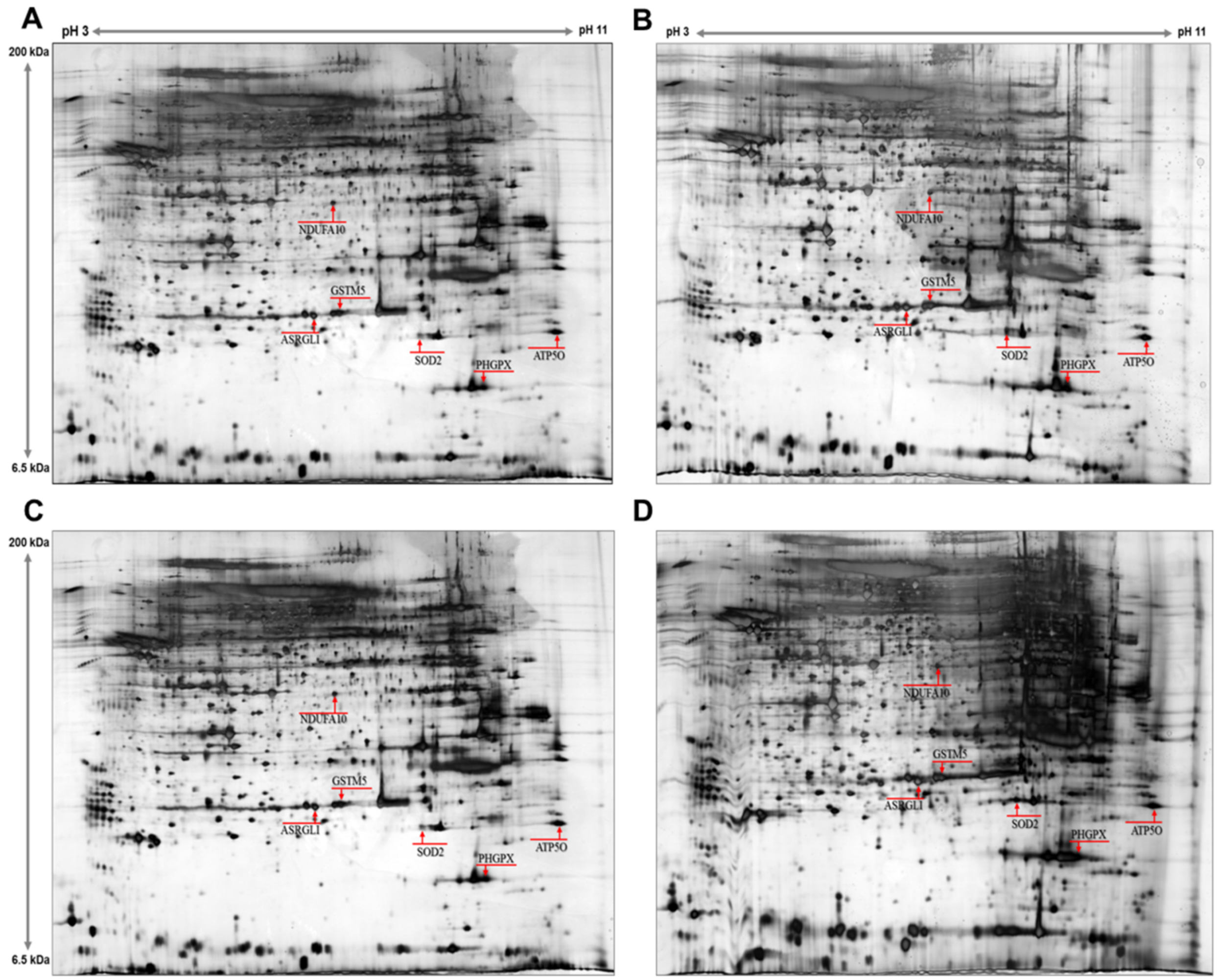

Figure S1. 2-DE protein spot patterns from adult spermatozoa. (*A*) Control (corn oil). (*B*) TDI treatment. (*C*) NOAEL treatment. (*D*) LOAEL treatment.
